# *Agave amica* a potential model for the study of agavins metabolism

**DOI:** 10.1038/s41598-023-47062-3

**Published:** 2023-11-14

**Authors:** Luis Francisco Salomé-Abarca, Ruth Esperanza Márquez-López, Mercedes G. López

**Affiliations:** 1 Departamento de Biotecnología y Bioquímica, Centro de Investigación y de Estudios Avanzados del IPN-Unidad Irapuato, 36824 Guanajuato, Mexico; 2 Instituto Politécnico Nacional, Centro Interdisciplinario de Investigación Para el Desarrollo Integral Regional-Unidad Oaxaca, 71230 Oaxaca, Mexico

**Keywords:** Plant sciences, Diseases, Chemistry

## Abstract

Fructans found in agave are called agavins, highly branched neo-fructans. They are essential on the yield and quality of Tequila production. The need for agave specimens with higher accumulation of agavins became essential before the growing demand of such products. To get such specimens, understanding agavins metabolism is a quintessential requirement. For this, a more efficient biological model is required. The recently reclassified *Agave amica* possesses the potential to gather the requirements for becoming such a model. Therefore, this study dealt with the characterization of carbohydrates in the bulbs of *A. amica* focusing on fructans. Moreover, it tested and described its feasibility as model for the accelerated study of agavins. Infrared analysis unveiled potential content of fructans in the bulbs of *A. amica*. Furthermore, high performance thin layer chromatography detected fructooligosaccharides. High performance anion exchange chromatography confirmed a polydisperse mixture of branched fructans. Gas chromatography–mass spectrometry analysis demonstrated agavins like structures in the bulbs of *A. amica*. Moreover, total fructan content and multivariate data analysis through bulb’s age demonstrated their correlation. Thus, the presence of agavins, their correlation with phenology, and their technical advantages highlighted the feasibility of this species as a potential new biological model for the study of agavins’ metabolism.

## Introduction

Fructans are present in several organisms except in those from animalia^1^. They are believed to play physiological roles in their bearing species as carbohydrate reservoir^2^. Fructans have also been hypothesized as an adaptative response to abiotic stress in cold, hot, and dry environments^1,3,4^. Some of the most popular fructan bearing specimens are chicory, onion, pachysandra, ryegrass, wheat, and agave^1^. Chemically, fructans are defined as non-structural carbohydrates differentiated from other oligo and polysaccharides because of their main fructose composition; if any, there is only one terminal (inulin) or internal (*neo*) glucose in their structures^5^.

Therefore, their chemical moieties possess *β*(2 → 1) and/or *β*(2 → 6) configuration^5,6^. These molecules possess wide structural diversity, they can be found as linear structures containing glucose and fructose (inulin and levan), or only fructose (F-series), *neo*-fructans, branched graminans, and highly branched *neo*-fructans, the so called agavins^7,8^. Between them, agavins possess the highest isomer rate among fructans. The structural complexity of agavins might be correlated with their multiple usages in pharmacy and food industry^9–12^. Furthermore, agavins are directly correlated to the yield and quality of traditional distillated beverages in Mexico such as Tequila and Mezcal^13^. These iconic beverages, production and quality, depend completely on the accumulation of agavins, even if that might take between 6 and 8 years in the field. However, regardless of their versality, agavins have not been fully exploited due to the lack of the full understanding of their metabolism. Additionally, they have been found only in commercial species of *Agave* and *Dasylirion*^7^. This fact makes these species unique biological models for their characterization at chemical, biochemical, and molecular level.

Nonetheless, *Agave* and *Dasylirion* specimens possess several operative disadvantages as biological models. Taken as example, the large size in mid to old specimens (3–8 years), which difficult their sampling, transport, and green house experiments. This parallel carries with some other limitations such as the possibility of controlling environmental factors to determine their effects over agavins synthesis. The same goes for enzyme studies, which need fast transport of biological material to laboratory^14^. Thus, the need for a simpler model in the study and characterization of agavins’ metabolism arises.

In this regard, the tuberose plant, previously known as *Polianthes tuberosa*, was reclassified as *Agave amica* (Medik.) Thiede and Govaerts included in Agavoideae^15^. As *Agave tequilana* Weber and all former *Agave* species, *A. amica* belongs to the native flora of Mexico. The tuberose was cultivated by Aztecs as ornamental and cut flower, and its name was “Omixochitl”, which means the bone flower. Nowadays, the tuberose is commonly called “Nardo” (nard) and it is cultivated worldwide as cut flower^16^.

As its reclassification suggests, the tuberose plant shares more than its geographical origin with former *Agave* species. It possesses a true bulb, which functions as a reservoir organ^17^. Thus, in a broad sense, it can be compared to agave pines, which also serve as a reservoir organ^14^. Unfortunately, since most of metabolite research in *A. amica* has been focused on volatiles from their flowers, little can be said about metabolic similarities with former *Agave* species^18–20^. Nevertheless, metabolite scrutiny of in vitro produced *A. amica* showed its potential to synthesize exopolysaccharides. Anyhow, differently from fructans, they are composed of glucuronic acid and other carbohydrates^21,22^. However, the inner polysaccharide reserve of the bulbs of *A. amica* has never been explored. Thus, its characterization targeting simple sugars and polysaccharides could reinforce the closeness between *A. amica* and the former *Agave* species. Therefore, highlights its potential as a new biological model for its use (in green houses) as a new way to try to understand agavins metabolism and accumulation in *Agave* species, mainly those used to make Tequila end Mezcal, which are highly difficult to study since it takes years in the field to reach their commercial “maturation” level. Thus, the aims of this research were to determine the presence of agavins in the bulbs of *A. amica* and to describe its potential as a new biological model for the study of agavins’ synthesis.

## Results

### Fourier transform infrared (FT-IR) analysis unveiled potential fructan content in the bulbs of *Agave amica*

**Figure 1:**
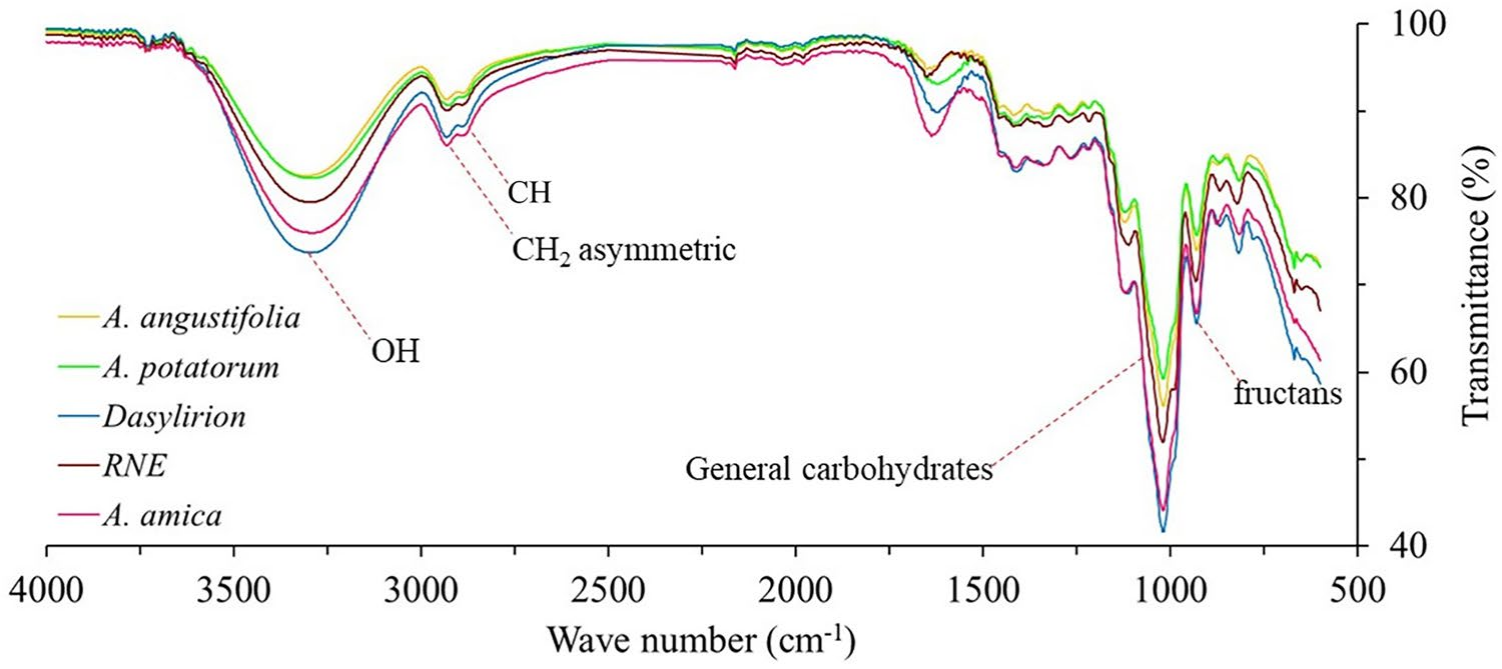
Diagnostic FT-IR bands of fructan extracts of *Agave amica, Agave angustifolia, Agave potatorum, Dasylirion* sp., and RNE.

The ATR-FT-IR analysis showed carbohydrate related bands along the medium infrared spectrum. For instance, a broad band around 3500 cm^−1^ produced by stretching vibrations of hydroxyl groups. The spectra also showed typical bands of asymmetric and symmetric stretching vibrations of CH and CH_2_ between 3000 and 2800 cm^−1^. In addition, strong carbohydrate transmittance bands were observed at 1250–900 cm^−1^ with a clear band between 950 and 920 cm^−1^, a characteristic fructan signal (Fig. 1). Moreover, the fructan fingerprinting region (1800–800 cm^−1^) of *A. amica* fitted well with those of former *Agaves*, *Dasylirion* sp., and raftiline (RNE). Thus, IR analysis strongly suggested the presence of fructans in the bulbs of *A. amica*.

### High performance thin layer chromatography (HPTLC) analysis confirmed fructooligosaccharides in the bulbs of* Agave amica*

**Figure 2:**
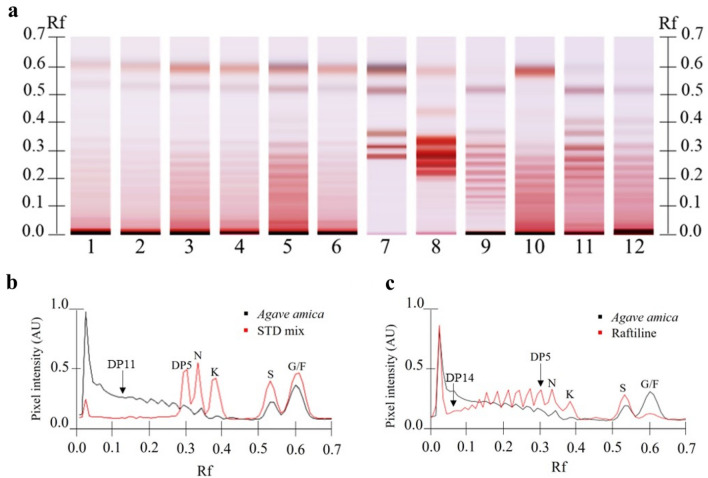
HPTLC analysis of fructooligosaccharides in the bulbs of *Agave amica*. (**a**), HPTLC plate with *A. amica* samples (lanes 1–6), standard compounds (lane 7), RSE (lane 8), RNE (lane 9), *Dasylirion* sp. (lane 10), *Agave angustifolia* (lane 11), and *Agave potatorum* (lane 12). The standard compound mixture (STD mix) contains fructose (F), glucose (G), sucrose (S), 1-kestose (K), 1-nystose (N), and 1-F fructofuranosylnystose (DP5) at 2 mg/mL. Standard compounds appear from top to bottom in the mentioned order. (**b**), Densitogram comparison between an *A. amica* sample and the standard mixture. (**c**), Densitogram comparison between *A. amica* and RNE.

The visual inspection of the HPTLC plates showed similar patterns between *A. amica* and those of the fructan containing *A. potatorum*, *A. angustifolia*, and *Dasylirion* sp. (Fig. 2). All profiles displayed pink and blue colored bands, typical of fructooligosaccharides, which sequentially decreased their R_f_ values as their polymerization degree (DP) increased (Fig. 2a). Nonetheless, the bulbs of *A. amica* (lanes 1–6) showed more content of fructose (R_f_ 0.57) than former agaves, but similar content to that of *Dasylirion* sp. In the case of sucrose (R_f_ 0.51), *A. amica* showed more content of this sugar than *Dasylirion* sp., but similar to those of former agaves. Moreover, the content of *neo*-kestose (R_f_ 0.40) was clearly variable among species in the next decreasing order: *A. potatorum* > *A. angustifolia* > *A. amica* > *Dasylirion* sp. (Fig. 2a). DP-11 (R_f_ 0.09) was the maximum visually countable DP for *A. amica* (Fig. 2b). This was corroborated by comparing densitograms of *A. amica* and RNE (Fig. 2c). Additionally, a strong coloration at the application point of *A. amica* lanes indicated accumulation of higher-DP fructans. This was also observed at the lanes of other fructans bearing controls (Fig. 2a).

**Figure 3:**
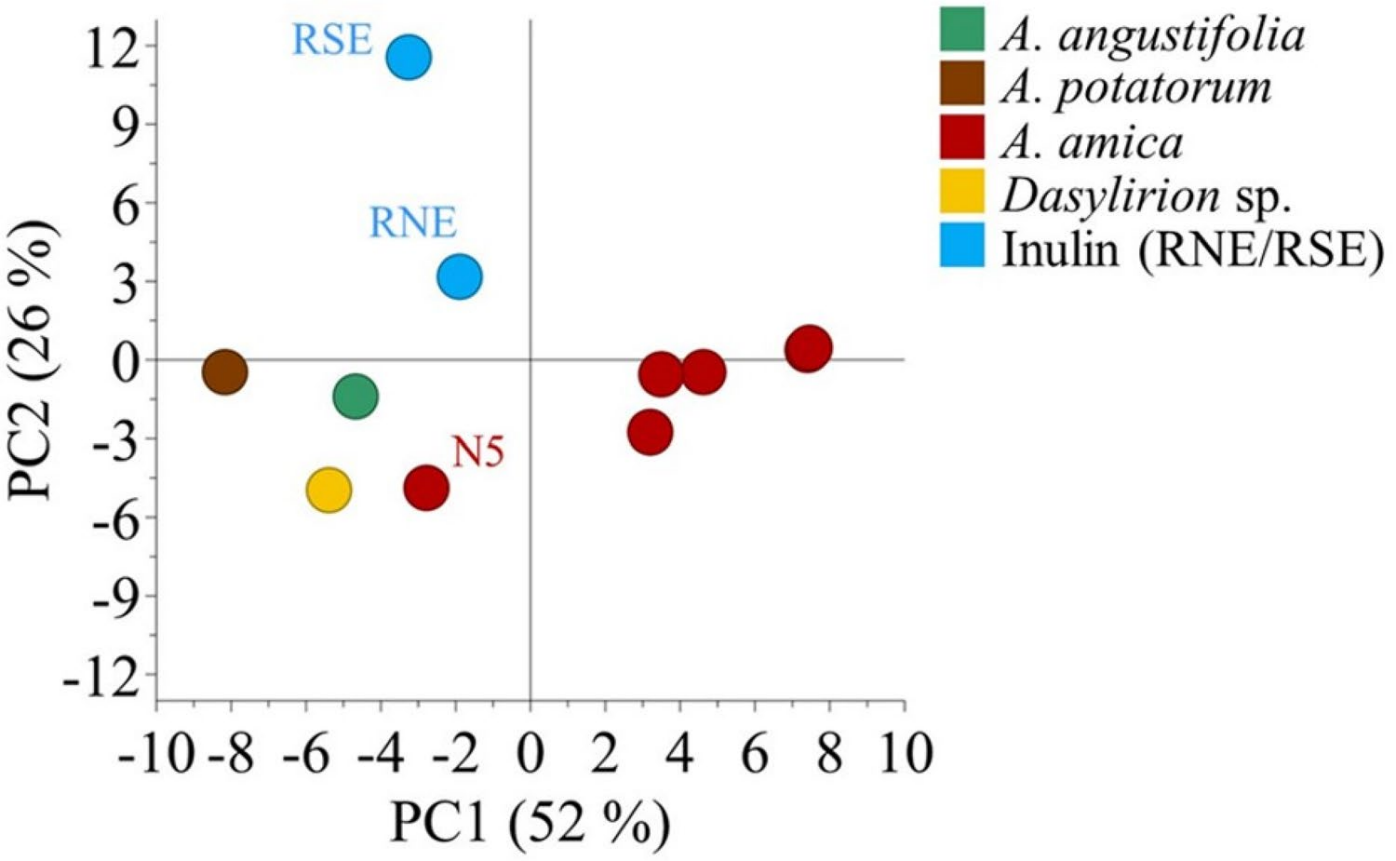
PCA of fructans from *Agave amica*, *Agave angustifolia*, *Agave potatorum*, *Dasylirion* sp., RNE, and RSE analyzed by HPTLC.

Furthermore, principal component analysis (PCA) of HPTLC data resulted in a model with 2 principal components (PCs), which explained around 80% of the total variation of the model (RX^2^*cum* = 0.78). The PC1 and PC2 captured 52% and 26% of such variation, respectively. The PCA score plot showed that *Agave* controls, *Dasylirion*, RNE, and one sample of *A. amica* (N5) were clustered together but separated from the rest of *A. amica* samples along the PC1. Conversely, the RSE control was separated along the PC2 far away from the other samples (Fig. 3). Thus, the HPTLC analysis demonstrated that *A. amica* bulbs possess fructans of low DP (fructooligosaccharides) plus a potential content of higher-DP polysaccharides. Nevertheless, HPTLC analysis did not provide information about fructan structure, that is, if they possessed linear or branched arrangement.

### High performance anion exchange chromatography-pulsed amperometric detection (HPAEC-PAD) determined branched fructans in the bulbs of* Agave amica*

**Figure 4:**
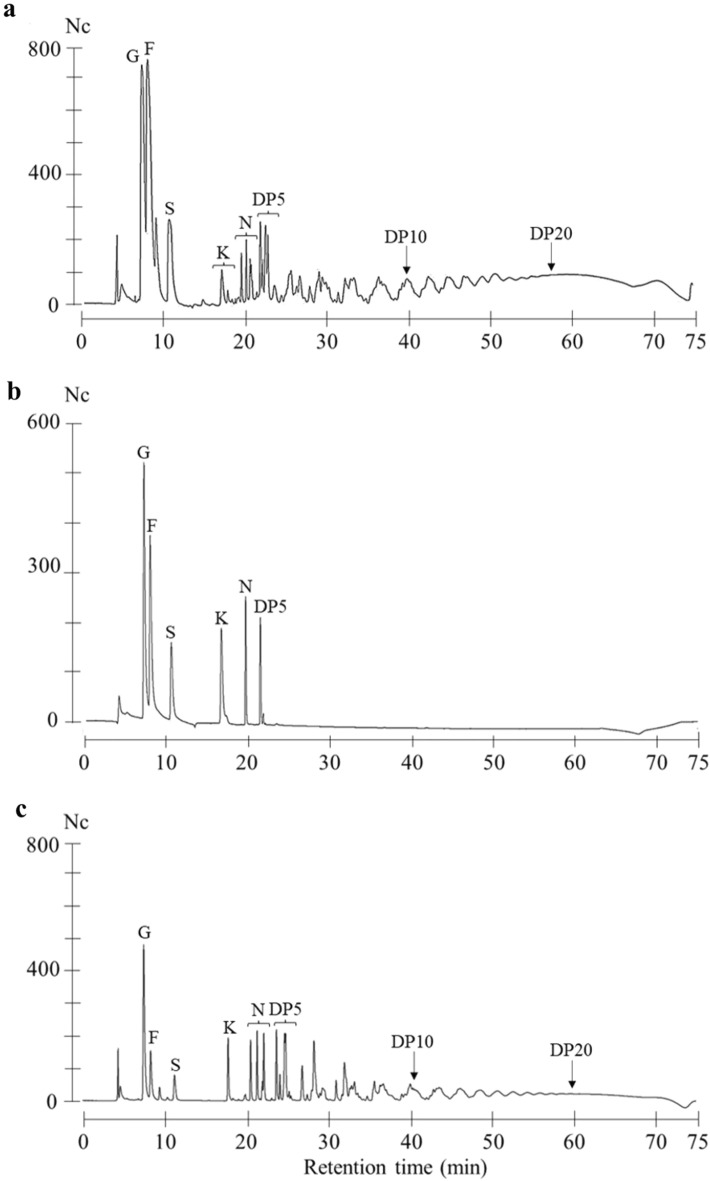
HPAEC-PAD analysis of branched fructans in bulbs of *Agave amica*. (**a**), HPAEC chromatogram of branched fructans in *A. amica*. (**b**), Chromatogram of standard compounds: glucose (G); fructose (F); 1-kestose (K); 1-nystose (N); 1-F fructofuranosylnystose (DP5) [12.5 µg/mL]. (**c**), Chromatogram of branched fructans in *Agave potatorum*. Peak intensities are expressed in nano Columbs (Nc).

To determine the general structural arrangement of fructans in the bulbs of *A. amica*, an HPAEC-PAD analysis was carried out in extracts of this species. *Agave potatorum* was used as branched fructan reference and RNE as linear fructan control. Thus, the analysis was expected to detect fructooligosaccharides, higher-DP fructans, and distinguish them between linear and branched. Sample examination detected glucose, fructose, sucrose, and a polydisperse mixture of fructans in the bulbs of *A. amica*. The HPAEC profile of *A. amica* showed a characteristic profile of branched fructans. In detail, several chromatographic peaks were detected for 1-kestose (DP-3), 1-nystose (DP-4), and 1-F fructofuranosylnystose (DP-5). Additionally, an irregular mountain like pattern was observed for higher-DP fructans (Fig. 4a,b) and the profile of *A. amica* resembled that of *A. potatorum* (Fig. 4c). Conversely, RNE-inulin showed symmetric HPAEC peaks (see Supplementary Fig. S1).

The countable polymerization degree for branched fructans in the bulbs of *A. amica* was DP-20 (Fig. 4a). This DP was determined using DP-5 standard as reference and RNE peaks to count from DP-6 onwards. Furthermore, a broad chromatographic peak was detected at the end of the HPAEC chromatogram; this peak contained all higher-DP fructans not resolved by the elution gradient. Thus, HPAEC-PAD analysis demonstrated that the bulbs of *A. amica* possesses branched fructans ranging from fructooligosaccharides to high-DP. Unfortunately, HPAEC-PAD was uncapable to distinguish between branched graminans and agavins.

### Glycosidic-linkage analysis demonstrated fructan like agavins in the bulbs of *Agave amica*

To get structural details of branched fructans of *A. amica*, glycosidic linkage analysis was performed by GC–MS. For this, partially methylated alditol acetate (PMAA)-derivatives from *A. amica*, *A. potatorum*, *A. angustifolia*, *Dasylirion* sp., and commercial *Dahlia* sp. linear inulin (high-DP) were analyzed. The analysis showed that *A. amica* possessed the same chromatographic PMAA profile of those of the agavins containing *A. potatorum*, *A. angustifolia*, and *Dasylirion* sp. In detail, eight chromatographic peaks were observed at 33.57, 33.83, 35.75, 40.88, 41.67, 41.99, 44.11, and 52.95 min (see Supplementary Fig. S2).

Furthermore, mass fragmentation patterns comparison between the glycosidic linkages of *A. amica* and other former *Agave* species further confirmed the presence of agavins. For instance, chromatographic peaks 1 and 2 (terminal fructose) of *A. amica* and agavin references showed characteristic mass pairs at *m/z* 161 and 162, *m/z* 145 and 146, *m/z* 101 and 102 from mannitol and glucitol epimers of *β*-d-fructofuranose (*t*-*β*-d-Fru*f*) (Table 1). PMAA-peak 3 indicated terminal *α*-d-glucopyranose (*t*-*α*-d-Glc*p*), and its spectrum was characterized by a base peak at *m/z* 102 and similar intensities between *m/z* 161 and 162 (33.8% and 31.6%, respectively). The *m/z* 118 and 205 were representatives of fragmentation between C2 and C3 next to methoxylated carbons. Peak 4 bore an indicative signal for acetylation in O6 at *m/z* 189. Higher intensity of *m/z* 162 over *m/z* 161 denoted an asymmetric effect caused by the reduction of methylated derivatives. This represented typical *β*(2 → 6) linkages in mannitol configured as 2–6-d-fructofuranose (*β*-2–6-d-Fru*f*).

**Table 1:** Linkage type and fragmentation pattern of PMAA derivatives of agavins in *A. amica* bulbs.

Peak	Rt^a^	Derivative	Linkage type	Fragmentation pattern (*m/z*)
1	33.5	2,5-di-O-acetyl-(2-deuterio)-1,3,4,6-tetra-O-methyl-d-mannitol	*t*-*β*-d-Fru*f*	129(100), 162(47.6), 161(30.4), 87(23.6), 101(13.6), 102(12.5), 75(10.5), 145(8.7), 72(7.5), 146(7.8)
2	33.8	2,5-di-O-acetyl-(2-deuterio)-1,3,4,6-tetra-O-methyl-d-glucitol	*t*-*β*-d-Fru*f*	129(100), 162(39.8), 161(35.6), 87(24.2), 101(13.9), 102(12.2), 75(10.3), 72(8.6), 146(6.7), 145(6.1)
3	35.7	1,5-di-O-acetyl-(1-deuterio)-2,3,4,6-tetra-O-thylglucitol	*t*-*α*-d-Glc*p*	102(100), 129(70.8), 145(55.2), 118(53.8), 101(51.1), 71(34.6), 87(35.8), 162(33.8), 161(31.6), 205(19.6), 72(1.68)
4	40.8	2,5,6-tri-O-acetyl-(2-deuterio)-1,3,4-tri-O-methylmannitol	(2 → 6)-*β*-d-Fru*f*	129(100), 162(41.2), 87(32.1), 189(16.0), 99(13.1), 102(11.9), 75(8.6), 72(6.35), 71(3.74), 60(2.11)
5	41.6	1,2,5-tri-O-acetyl-(2-deuterio)-3,4,6-tri-O-methylmannitol	(2 → 1)-*β*-d-Fru*f*	129 (100), 87 (30.1), 190 (23.3), 161 (22.1), 101 (10.9), 100 (10.4), 75 (6.9), 145 (6.6), 71 (6.4), 72 (5.2)
6	41.9	2,5,6-tri-O-acetyl-(2-deuterio)-1,3,4-tri-O-methylglucitol 1,2,5-tri-O-acetyl-(2-deuterio)-3,4,6-tri-O-methylglucitol	(2 → 6/1)-*β*-d-Fru*f*	129 (100), 87 (30.1), 161 (22.3), 190 (17.3), 162 (11.0), 101 (9.6), 100 (8.2), 189 (6.7), 71 (6.1), 75 (6.6), 72 (5.25), 118 (1.4)
7	44.1	1,5,6-tri-O-acetyl-(1-deuterio)-2,3,4-tri-O-methylglucitol	*i*-*α*-d-Glc*p*	102 (100), 118 (69.5), 129 (68.4), 87 (48.4), 101 (28.4), 162 (25.6), 189 (20.0), 71 (14.8), 233 (7.9), 145 (0.80)
8	52.9	1,2,5,6-tetra-O-acetyl-(2-deuterio)-3,4-di-O-methylhexitol	1,6-di-*β*-d-Fru*f*	129 (100), 87 (36.7), 190 (24.2), 189 (16.2), 99 (11.6), 100 (11.1), 72 (3.8), 71 (3.3), 60 (1.5)

^a^Rt, retention time expressed in minutes.

PMAA-peaks 4 and 5 (indicative of linkages *β*(2 → 1)), both epimers of mannitol were better separated than the epimers of glucitol, which were totally convoluted in the chromatographic peak 6. Because of this, peak 6 produced a fragmentation pattern containing *m/z* values for both *β*(2 → 1) and *β*(2 → 6) linkages (Table 1). Peak 7 demonstrated the presence of internal *α*-d-glucopyranose (*i*-*α*-d-Glc*p*), that is, *neo*-fructans. This was confirmed by its highly distinctive *m/z* 233, which indicated an extra acetylation at C6. Finally, peak 8 (1,6-di-*β*-d-Fru*f*) was an indicative of branching points in agavins (Table 1).

To propose chemical structures for fructans in *A. amica*, molar contribution data from PMAAs was used (Table 2). Thus, it suggested general structures of all fructans contained in the extracts, which open the possibility of other DPs and agavin conformations. Glycosidic linkages were classified and correlated to molecule types in the structures. The *t*-*β*-d-Fru*f* linkages suggest the number of terminal fructose units in the average structure; *t*-*α*-d-Glc*p* provides the proportion of terminal glucose (inulin type), and *i*-*α*-d-Glc*p* the proportion of internal glucose (*neo*-fructans). The ratio between them indicates the probability in which graminans and *neo*-fructans are found in the extract. The *β*-2-6-d-Fru*f* and *β*-2-1-d-Fru*f* linkages determined the number fructose with levan and inulin configuration, respectively. The linkage 1,6-di-*β*-d-Fru*f* indicated the number of branching points in the structure, and the ratio *β*-2-1-d-Fru*f*:1,6-di-*β*-d-Fru*f* suggested the branching frequency, that is, how many fructose units are between branches.

**Table 2:** Molar ratios of glucose/fructose types and molecule number assigned for each glycosidic linkage type in agavins from *Agave amica*.

Linkage type	N1	N2	N3	N4	N5	N6	Average	Stdev^a^
*t*-*β*-d-Fru*f*	1.8	2.3	2.0	2.0	2.0	2.0	2.0	0.16
*β*-2-1-d-Fru*f*	2.2	2.8	2.1	2.2	2.3	1.8	2.2	0.30
*β*-2-6-d-Fru*f*	0.3	0.3	0.2	0.3	0.2	0.4	0.3	0.06
*1*,6-di-*β*-d-Fru*f*	0.6	0.8	0.6	0.5	0.5	0.6	0.6	0.11
*α*-d-Glc*p*	1.0	1.0	1.0	1.0	1.0	1.0	1.0	1.25
Core-DP	5.9	7.2	5.9	6.0	6.0	5.8	6.1	0.53

	Assigned molecules per linkage type							
*t*-*β*-d-Fru*f*	2	2	2	2	2	2	2.0	0.00
*β*-2-1-d-Fru*f*	2	3	2	2	2	2	2.2	0.37
*β*-2-6-d-Fru*f*	1	1	1	1	1	1	1.0	0.00
*1*,6-di-*β*-d-Fru*f*	1	1	1	1	1	1	1.0	0.00
*α*-d-Glc*p*	1	1	1	1	1	1	1.0	0.00
DP	7	8	7	7	7	7	7.2	0.37

^a^Standard deviation.

As there can exist only one glucose in a fructan structure, a ratio between the molar percentage of glucose and the other units was calculated. The ratio was determined by dividing the molar percentage of each linkage by the sum of terminal and internal glucose. The sum of molar ratios determined the DP value for each sample. An average was calculated from values of all samples (n = 6). Subsequently, a molecule number was assigned according to the obtained values (Table 2). Any value higher than zero but lower than one was considered as one molecule. For values with decimals < 0.5 the molecule number was truncated, and ratios with decimal values ≥ 0.5 were rounded to a full number. The resulting values obtained from these calculations were arranged to a minimum core structure needed for a graminan and agavin. Furthermore, the polymerization degree determined by HPAEC was divided by the previously calculated average DP value. Thus, molecule values, except glucose, were multiplied for this product to suggest larger structures.

**Figure 5:**
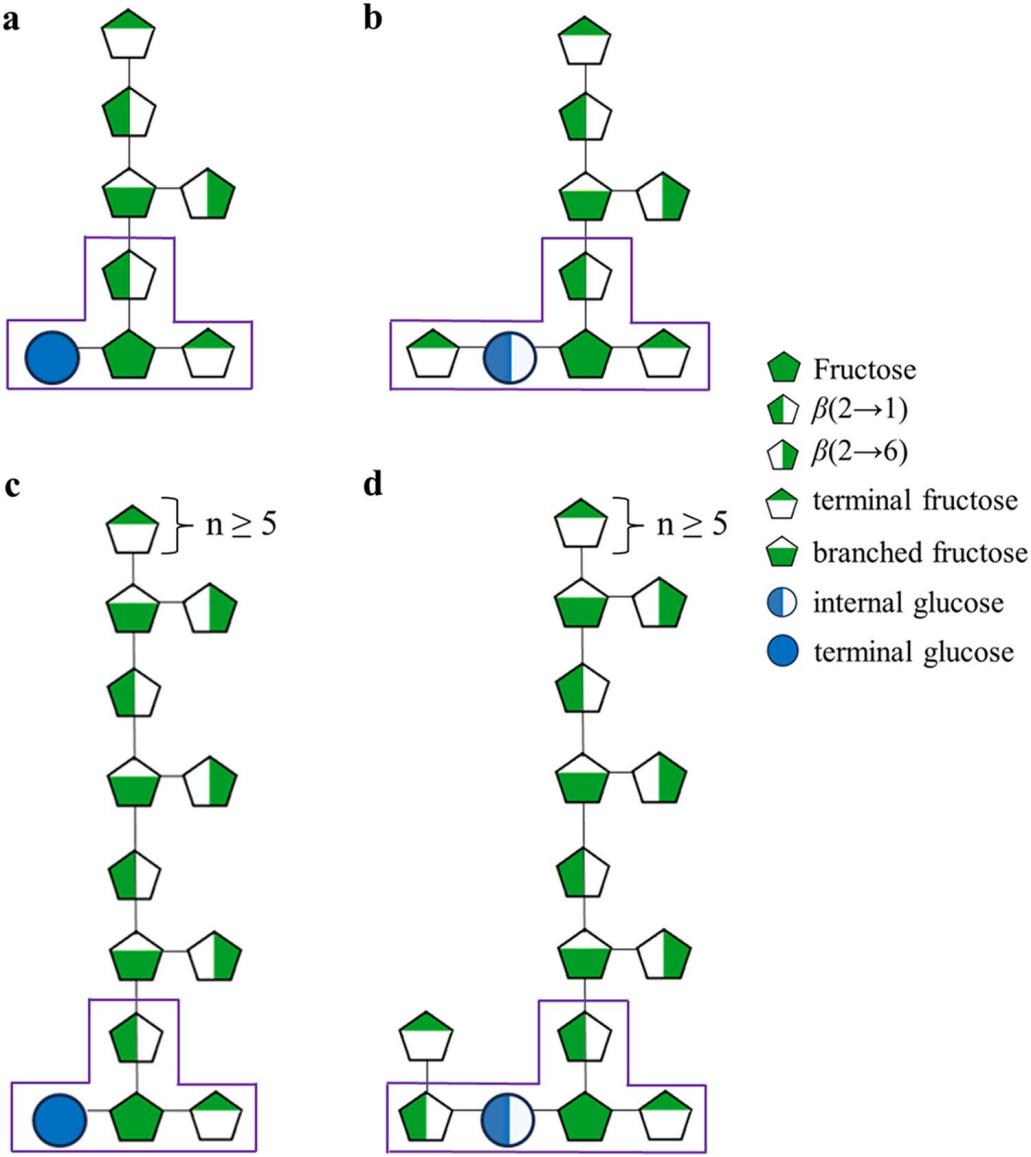
Proposed chemical structures for agavins contained in the bulbs of *Agave amica*. (**a**), average graminan structure in the bulbs of *A. amica*. (**b**), average agavin structure in the bulbs of *A. amica*. (**c**), average proposed structure for a DP-20 graminan in *A. amica*. (**d**), average proposed structure for a DP-20 agavin in *A. amica*. Carbohydrate units inside a purple frame constitute a core structure, which indicates the minimum construction block needed for the smallest graminan or agavin to which all other units are arranged. The term n ≥ indicates that the marked part of the structure can be at least 5 fructose (inulin) larger to reach a DP-20 or higher.

Thus, a 1:1 ratio between *t*-*α*-d-Glc*p* and *i*-*α*-d-Glc*p* indicated that fructan mixture in *A. amica* is composed of the same proportion of graminans and *neo*-fructans. A higher proportion of *β*(2 → 1) over *β*(2 → 6) indicated that the general structure of agavins in *A. amica* consisted mainly of inulin chains with short levan branches. Besides, the proportion of *β*-2-1-d-Fru*f* linkages (2.3) per each 1,6-di-*β*-d-Fru*f* moiety indicated that there is a branching point every two *β*-2-1-d-Fru*f* linkages (two fructose units) in the inulin chain of *A. amica* agavins (Fig. 5). Thus far, *A. amica* accomplished the first quintessential requirement to be pointed as a highly potential alternative biological model to agave plants, that is, possessing agavins.

### *Agave amica* bulbs as potential model for agavin synthesis and accumulation through time

A second requirement for a good alternative biological model is showing variation of total fructans, agavins production, and accumulation trough time as occurs in commercial agaves. Thus, the previously analyzed one-year-old bulbs of *A. amica* were compared to 6 month and 2-years-old bulbs of the same species. The comparison was done in terms of total fructan content and agavin variation related to bulb’s age. The total fructan content showed an increment from 6 months to 1 year but a decrease in two-year-old specimens (see Supplementary Fig. S3).

**Figure 6:**
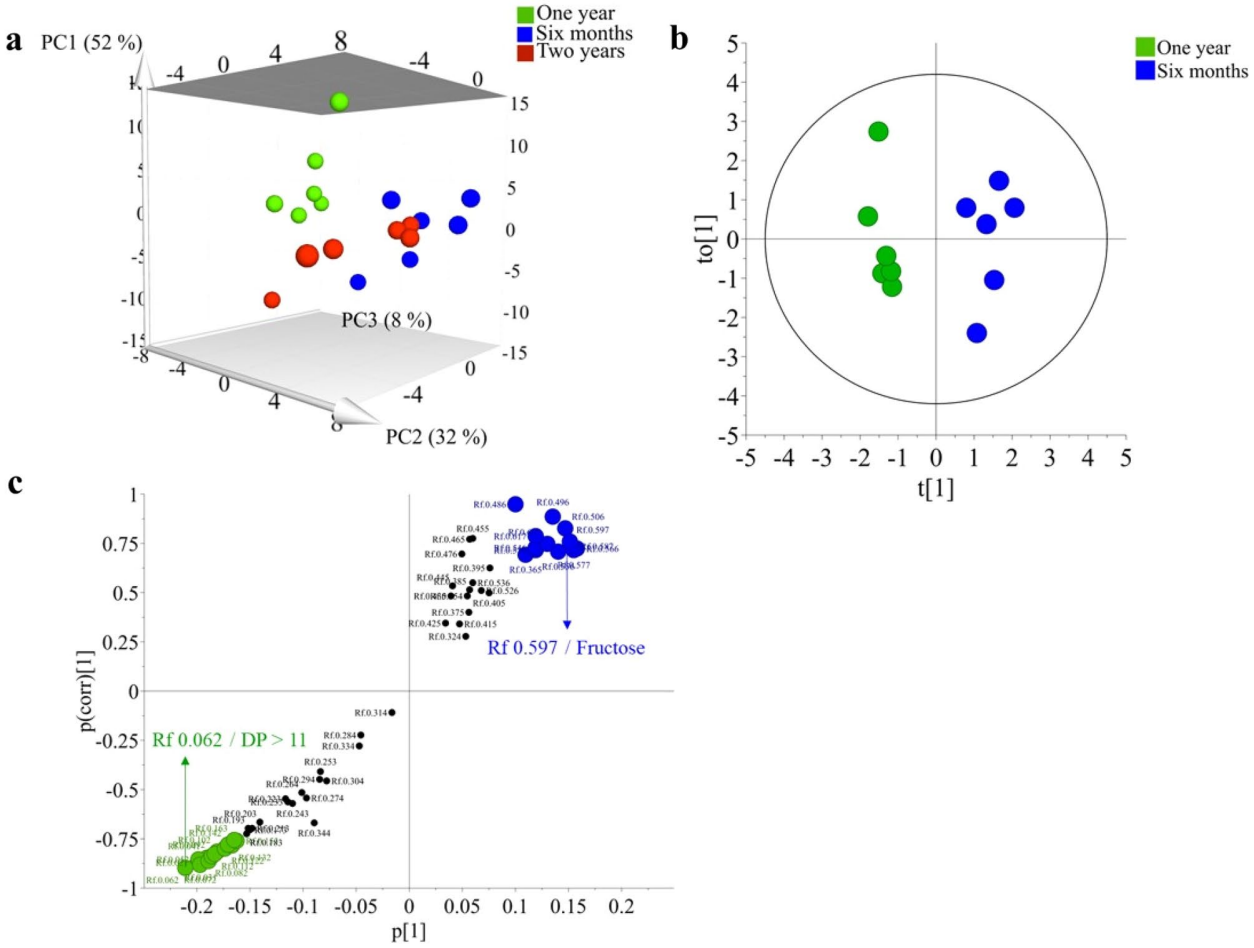
Multivariate analysis of HPTLC data from *Agave amica* bulbs of different ages. (**a**), PCA of *A. amica* bulbs of different ages. (**b**), OPLS-DA-model of agavins variation in 6 months and one-year-old bulbs of *A. amica*. (**c**), S-plot of OPLS-DA model for agavin variation in 6 months and one-year-old bulbs of *A. amica*.

The PCA-analysis of these bulbs confirmed that they possess differential agavin profiles (Fig. 6a). Subsequently, an orthogonal projection to latent structures-discriminant analysis (OPLS-DA) was performed on 6 month and one-year-old bulbs using their age as classes (Fig. 6b). The model was well validated (*Q*^*2*^ = 0.93, *p* = 0.0003) and the S-plot indicated that younger specimens are richer in glucose, fructose, and sucrose, while older specimens are richer in higher-DP fructooligosaccharides (R_f_ 0.062–0.163) (Fig. 6c). This corroborates the effect of age in the production of agavins in the bulbs of *A. amica*.

## Discussion

Before the growing demand of alcoholic beverages and prebiotics (fructans) from agave specimens, the need for a more practical biological model for the understanding of agavins metabolism and their accumulation through time is imperative. In this regard, recently, the genera *Polianthes*, *Manfreda*, and *Prochnyanthes* were added to the genus *Agave*^15^. In the former *Polianthes*, which all species are endemic to Mexico, the most recognized species is the tuberose plant^16^. Nowadays, the tuberose is cultivated worldwide as an ornamental or cut flower^17^*.* However, this species possesses a bulb and the capability of producing exopolysaccharides^22^. Therefore, its potential to produce and accumulate fructans was worth exploring.

The first characterization made by FT-IR analysis showed an OH band around 3500 cm^−1^ attributable to OH in carbohydrates. Nonetheless, this band is not specific for carbohydrates since it can be also produced by OH attached to polyphenols^23^. However, CH and CH_2_ bands detected between 3000 and 2800 cm^−1^ have been associated with polysaccharide cores^24^. Additionally, the strong carbohydrate bands between 1250 and 900 cm^−1^ indicated C–C and C–O stretching, and C–O–H and C–O–C bending vibrations of oligo- and polysaccharides^24,25^. Finally, a band between 950 and 920 cm^−1^ was diagnostic for fructans, previously detected in other fructan bearing species such as *A. potatorum* and *A. angustifolia*^24^. This band was also detected in *Dasylirion* sp. and RNE reinforcing the fructans’ specificity of this band.

The characterization of *A. amica* fructan by HPTLC showed a pink/blue spot pattern, which is characteristic for aldose/ketose components. That is, glucose -containing fructan will turn on blue spots after derivatization with aniline, and F-series will color pink instead^26^. This pattern and the decreasing R_f_ of spots as their DP increases have been specifically documented in fructan containing species such as *A. tequilana*, *A. potatorum*, *A. angustifolia*, *Agave fourcroydes*, *Cichorium intybus*, among others^7,14,27^. The maximum visible DP for *A. amica* was DP-11, which has been considered by some authors as the maximum DP of fructooligosaccharides^28^. Nonetheless, the strong reactivity of the application point on the HPTLC plate of *A. amica* and the other reference tracks to aniline suggested accumulation of higher-DP fructans^14^. Conversely, commercial raftilose (RSE) showed low reaction to aniline at low R_f_ values, which might be explained by its chemical nature itself. Since RSE is an hydrolyzation product of RNE, high-DP fructans are converted to lower-DP fructans causing lower coloration at the application point of this sample (Fig. 2a).

The HPTLC-PCA results showed a separation between RSE and the other fructan containing samples. This separation was caused by qualitative and quantitative differences among samples. That is, RSE did not possess some bands at the R_f_ 0.01–0.20 range or they were just faint bands (Fig. 2A). Additionally, the separation of *A. amica* from other agave references and RNE might be caused by differences in simpler sugars and/or quantitative differences on fructooligosaccharides, probably given by species and/or developing stage factors. Unfortunately, HPTLC did not provide information about fructans structures. However, *A. amica* fructans were determined as branched by HPAEC-PAD analysis. Fructans showed a typical irregular chromatographic peak profile of branched fructans observed in *Agave* spp. and *Dasylirion* sp.^7,14,24^. This pattern denoted the high isomeric rate caused by the increase of ramifications in higher-DP fructans^14,24^. The opposite has been observed for linear inulin that produces symmetric HPAEC-peaks, as occurs in chicory^29^. The broad peak at the end of the chromatographic elution indicated the presence of branched fructans with DP higher than 20. This chromatographic trend was confirmed by enzymatic hydrolysis of *A. angustifolia* fructans which resulted in the progressive reduction of such chromatographic peak^30^. Thus, *A. amica* possess the potential to produce high-DP fructans as *A. angustifolia.*

The characterization of sugar linkages in the fructans of *A. amica* showed the typical PMAA-pattern of agavin bearing species^7,14,24,31^. For instance, peak 4 (2 → 6)-*β*-d-Fru*f*) and 8 (1,6-di-*β*-d-Fru*f*) proved the presence of levan branches in the analyzed fructans. As an example, onion’s *neo*-inulin lacks these chromatographic peaks as they do not possess branches^1,7,31^. Furthermore, PMAA-peak 7 confirmed neo-glucose in fructans of *A. amica*^7,14^, which distinguishes agavins from graminans^7^. That is, graminans do not produce PMAA-peak 7. Additionally, the proposed chemical structures for agavins in *A. amica* were quite like those proposed for *A. tequilana* between six and seven-years-old^14^.

Moreover, PMAA analysis must be interpreted beyond a structural analysis since each linkage type could be associated to enzymatic activity^32^. For instance, the presence of *β*(2 → 1) linkages (PMAA-peak 5) suggested the activity of 1-fructosyltransferase in *A. amica*. Further, *β*(2 → 6)linkages (PMAA-peak 4) indicated the potential activity of sucrose:fructan 6-fructosyltransferase. Additionally, the differentiation between F-series and glucose-containing fructans observed by HPTLC and PMAA profiles suggested the activity of 6G-fructosyltransferase. Such an enzyme is responsible for adding glucose to a fructan either on graminans or *neo*-fructans^33^.

Thus far, the presence of agavins in the bulbs of *A. amica* has been demonstrated. In addition, total fructan content and agavins variation through age suggested an enzymatic dynamic like that of former *Agave* spp.^14^. Moreover, a biomass accumulation period of *A. amica* lasts around 1 year^34^, which considerably reduces the time needed for studying the kinetics of agavins production compared with other *Agave* species (6–7 years)^14^. Further, the possibility of growing *A. amica* under nursery conditions^34^ will allow for experiments under controlled conditions including time, pH, temperature, drought, among others, which are difficult to control in the field and follow them for years. Using *A. amica* as a model in such experiments will contribute to understand factors governing agavins content potentially extrapolated to commercially important *Agave* species. This might be of interest for agaves used to produce traditional alcoholic beverages such as tequila, mezcal, and pulque^13^, as well as agavins as prebiotics. In conclusion, *A. amica* has shown the potential needed for a good alternative biological model, which could accelerate the study and understanding of agavins metabolism and accumulation. Nevertheless, we should consider that additional genetic characterization must be carried out. Taken as an example, the detection and characterization of fructan synthesis related enzyme genes in *A. amica* such as fructosyltransferases among others. All these information may have an impact on commercially important *Agave* species related to Tequila and Mezcal quality and production, as well as in the manufacturing of agavins as prebiotics.

## Materials and methods

### Plant material

Samples consisted of double pearl bulbs of *Agave amica* (Medik.) Thiede & Govaerts commercially obtained from a local producer at Jalisco, Mexico (N 21° 13′ 54.9474″, W–102° 20′ 32.676″). The age of the bulbs was 6 months without sprouts, 1 year at the beginning of sprouting, and 2 years after flowering. The *A. amica* bulbs were removed with surrounding soil and transported in pots to the laboratory for later analysis. Specimens of *Agave potatorum*, *Agave angustifolia*, and *Dasylirion* sp. were used as agavin bearing reference samples. The plant study complies with relevant institutional, national, and international guidelines and legislation*.*

### Sample preparation

Total fructans were extracted from fibers with ethanol [80%] in a 1:1 ratio for *A. potatorum*, *A. angustifolia*, and *Dasylirion* sp. samples; for *A. amica* the extraction ratio was 2 mL of solvent per 1 g of fresh bulb material. The rest of the extraction procedure was performed as previously reported^7,35^.

### Fourier-transform infrared (FT-IR) spectroscopy

This analysis was carried out in a Cary 600 FT-IR spectrometer (Agilent Technologies, Folsom, CA, USA) equipped with an attenuated total reflectance (ATR) accessory and a diamond/Ge crystal plate (Agilent Technologies, Folsom, CA, USA) (MIRacle by PIKE Technologies, USA). Acquisition data parameters were those previously reported for agave fructan characterization^24^.

### High-performance thin layer chromatography (HPTLC)

Fructan extracts, 5 mg, were firstly dissolved in 300 μL of distilled water and then taken up to 1 mL with 700 μL of absolute ethanol. The samples were softly mixed and ultra-sonicated for two minutes. Samples were separated on silica gel HPTLC plates (20 × 10 cm, F_254_) purchased from Merck (Darmstadt, Germany). The analysis was performed in a CAMAG-HPTLC system (Muttenz, Switzerland). Conditions for sample analysis were those previously reported for fructooligosaccharide analysis^32^.

### High performance anion exchange chromatography-pulsed amperometric detection (HPAEC-PAD)

The fructan profiles of all specimens were performed in a liquid ion-exchange chromatograph DIONEX ICS-3000 (Thermoscientific, Waltham, MA, USA). The system was equipped with a Carbopac PA-200 (40 × 250 mm) column and a precolumn (40 × 25 mm). Elution and detection parameters were those previously reported for fructan analysis^32^. Standards of glucose, fructose, sucrose, 1-kestose, 1-nystose, and 1-F fructofuranosylnystose [12.5 µg/mL] were injected as reference compounds.

### Partially methylated alditol acetate (PMAA) derivatization

Ten milligrams of fructans of each plant material were individually dissolved in 500 µL of dimethyl sulfoxide and stirred overnight until complete dissolution. Derivatization to PMAAs was performed as previously described for glycosidic linkage analysis of agave samples^7^.

### Gas Chromatography coupled to mass spectrometry (GC–MS)

A system equipped with an Agilent technologies chromatograph (7890B), an automatic liquid autosampler (7683B), and a selective mass detector (5977A) was employed for GC–MS analysis. Samples were separated in a HP5-MS column (30 m × 0.25 mm × 0.25 µm). All analysis conditions were those reported for sugar-linkage analysis in agave specimens^7^.

### Total fructan content

Quantification was performed with the commercial K-FRUC kit (Megazyme International, Ireland) following the manufacturer instructions. Data was acquired at 410 nm in a spectrophotometer (Varian Cary 50 Bio).

### Data extraction and multivariate data analysis

Chromatographic images were processed with the software rTLC (Version1.0)^36^. Data extraction parameters were those previously reported for agave analysis^32^. Data was scrutinized by principal component analysis (PCA) using the Pareto scaling method. To characterize age affects, an orthogonal projection to latent structures discriminant analysis (OPLS-DA) was performed setting bulb’s age as classes. The OPLS-DA model was validated by permutation test (100 permutations) and CV-ANOVA test considering a *Q*^*2*^ ≥ 0.40 and *p *value ≤ 0.05, respectively. The discriminant R_f_ values were determined with an S-plot.

### Supplementary Information

Supplementary Figures.

## Data Availability

Data is available upon request to the corresponding author.
