# Author Correction: *Agave amica* a potential model for the study of agavins metabolism

**DOI:** 10.1038/s41598-024-55346-5

**Published:** 2024-02-27

**Authors:** Luis Francisco Salomé-Abarca, Ruth Esperanza Márquez-López, Mercedes G. López

**Affiliations:** 1 Departamento de Biotecnología y Bioquímica, Centro de Investigación y de Estudios Avanzados del IPN-Unidad Irapuato, 36824 Guanajuato, Mexico; 2 Instituto Politécnico Nacional, Centro Interdisciplinario de Investigación Para el Desarrollo Integral Regional-Unidad Oaxaca, 71230 Oaxaca, Mexico

Correction to: *Scientific Reports* 10.1038/s41598-023-47062-3, published online 14 November 2023

The original version of this Article contained a repeated error, where “CH_3_” was used instead of “CH” in relation to agavins infrared bands. The error has been corrected in the Results section, under the subheading ‘Fourier transform infrared (FT-IR) analysis unveiled potential fructan content in the bulbs of Agave amica’, in Figure 1 and in the Discussion section.

The original Figure 1 and accompanying legend appear below.

**Figure 1:**
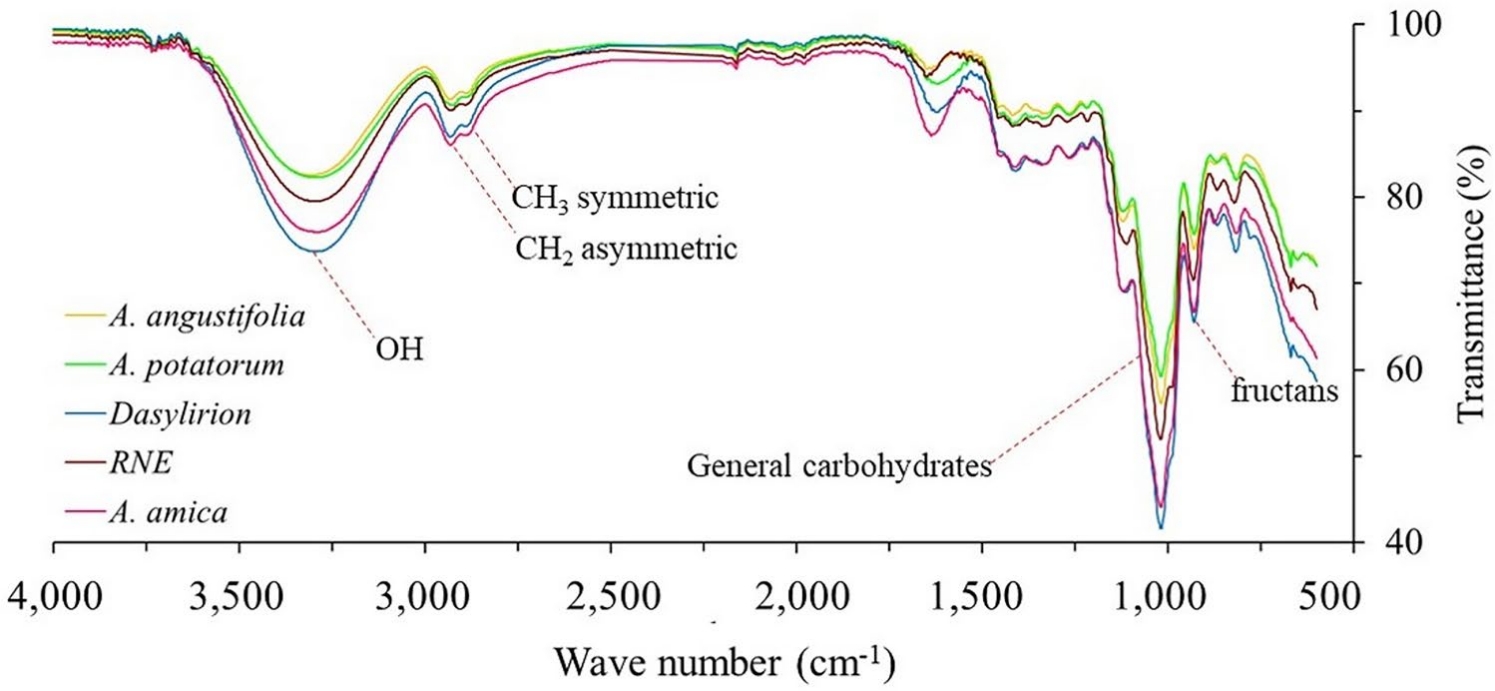
Diagnostic FT-IR bands of fructan extracts of *Agave amica*, *Agave angustifolia*, *Agave potatorum*, *Dasylirion* sp., and RNE.

The original Article has been corrected.

