# Supplementary material for: Agave amica a potential model for the study of agavins metabolism
Source: Sci Rep. 2023 Nov 14;13:19888. doi: 10.1038/s41598-023-47062-3 (PMC10645838; doi:10.1038/s41598-023-47062-3)
Supplement: Supplementary file 1 — Supplementary Figures. [file 41598_2023_47062_MOESM1_ESM.pdf]

Supplementary material

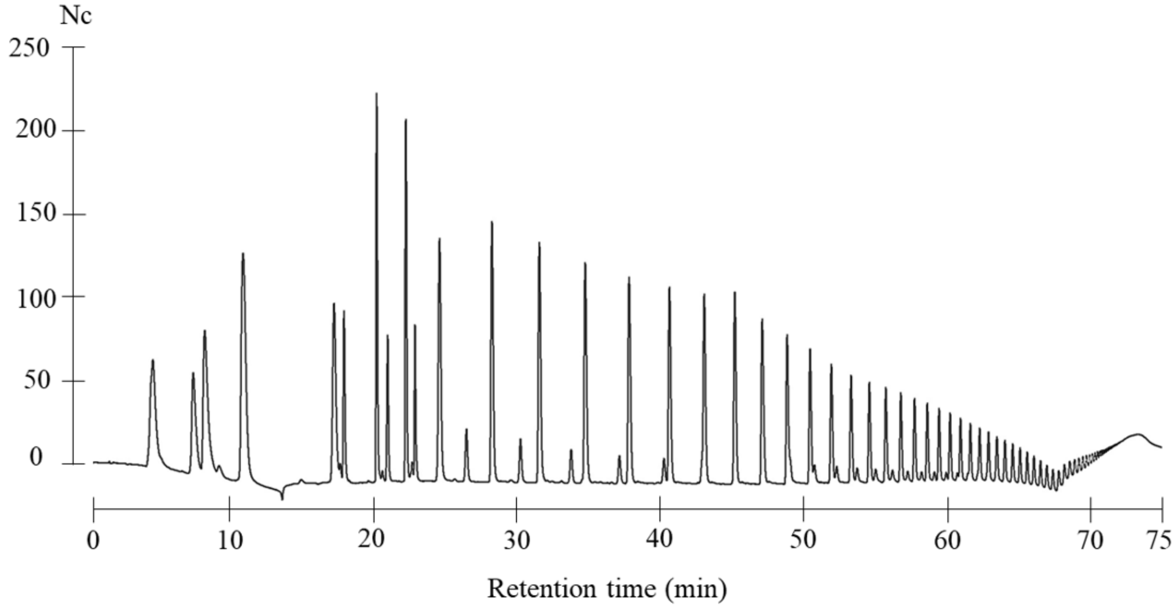

Supplementary Figure S1. HPAEC-PAD chromatogram of a linear inulin reference sample (RNE) from Beneo-Orafti. The HPAEC profile shows typical symmetric peaks of linear fructans.

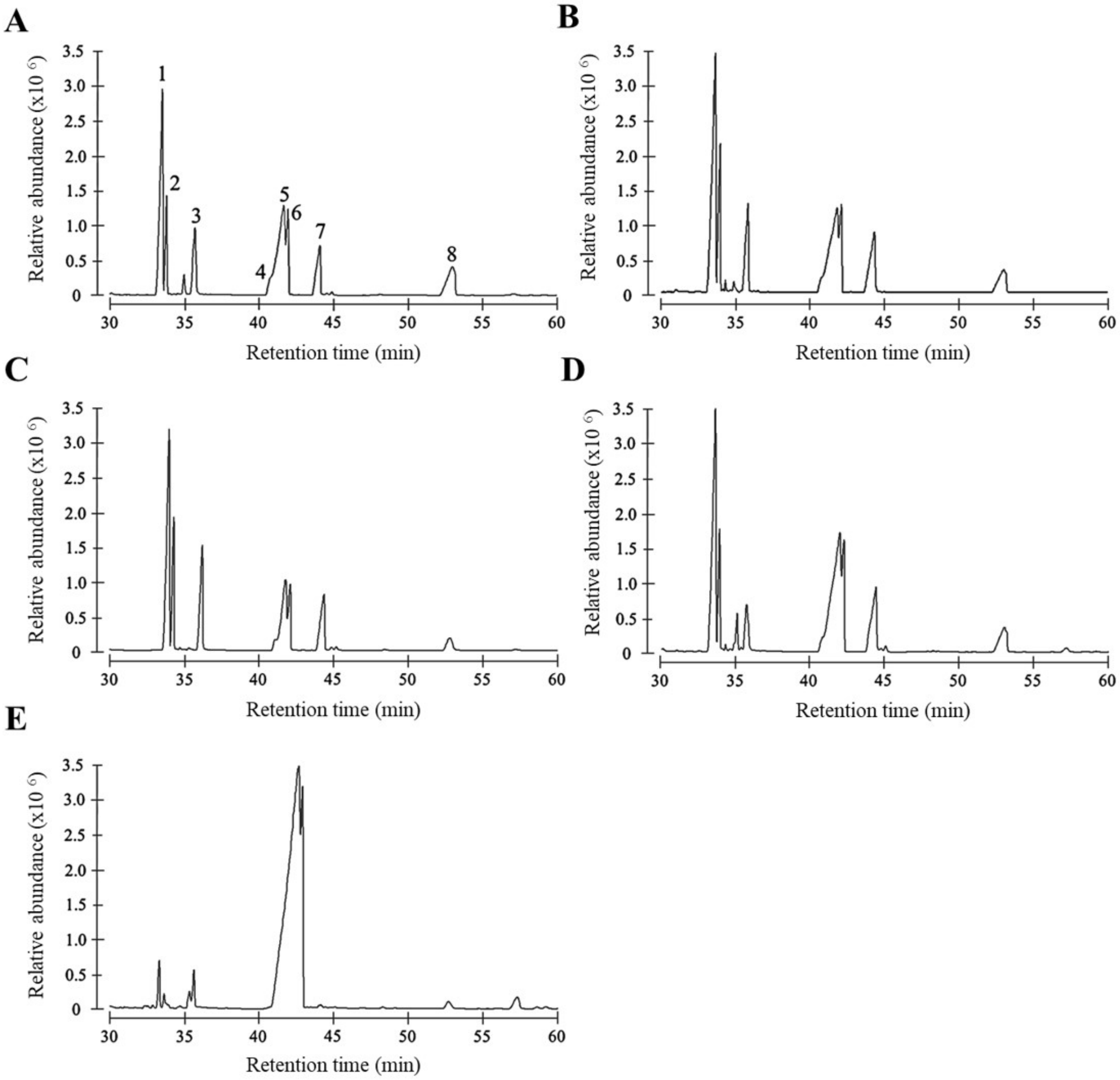

Supplementary Figure S2. Gas chromatography coupled to mass spectrometry analysis of partially methylated alditol acetate (PMAA) derivatives in **A,** *Agave amica*; **B**, *Agave angustifolia*; **C**, *Agave potatorum*; **D**, *Dasylirion* sp.; **E**, *Dahlia* inulin. 1, *β*-2-6-D-Fru*f*; 2, *β*-2-6-D-Fru*f*; 3, *t*-*α*-D-Glc*p*; 4, *β*-2-6-D-Fru*f*; 5, *β*-2-1-D-Fru*f*; 6, *β*-2-1-D-Fru*f*/*β*-2-6-D-Fru*f*; 7, *i*-*α*-D-Glc*p*; 8, 1,6-di-*β*-D-Fru*f*.

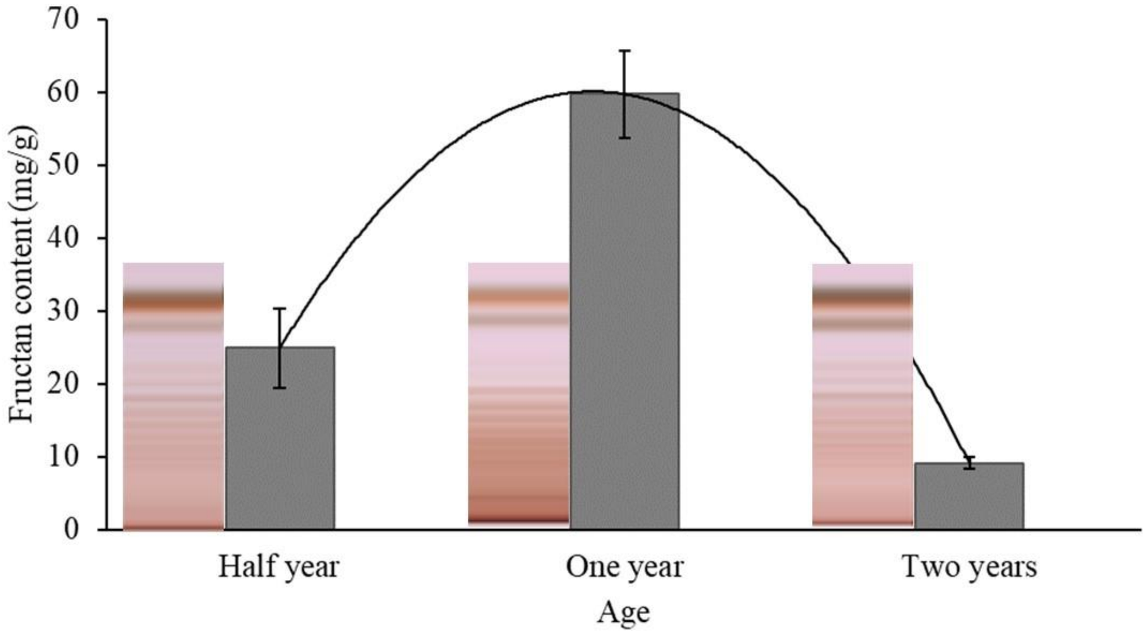

Supplementary Figure S3. Total fructan content in bulbs of *Agave amica* through age. Gray boxes represent average values (n = 6), and vertical bars indicate standard error. Pink boxes are representative HPTLC chromatograms of each corresponding bulbs' age. The image shows an increase and decrease of total fructan as the age of the specimens increments. The chromatograms show more content of simple sugars (top chromatographic bands) in half-year and two-year-old specimens. Moreover, it shows more content of fructooligosaccharides in one-year-old bulbs (lower chromatographic bands).

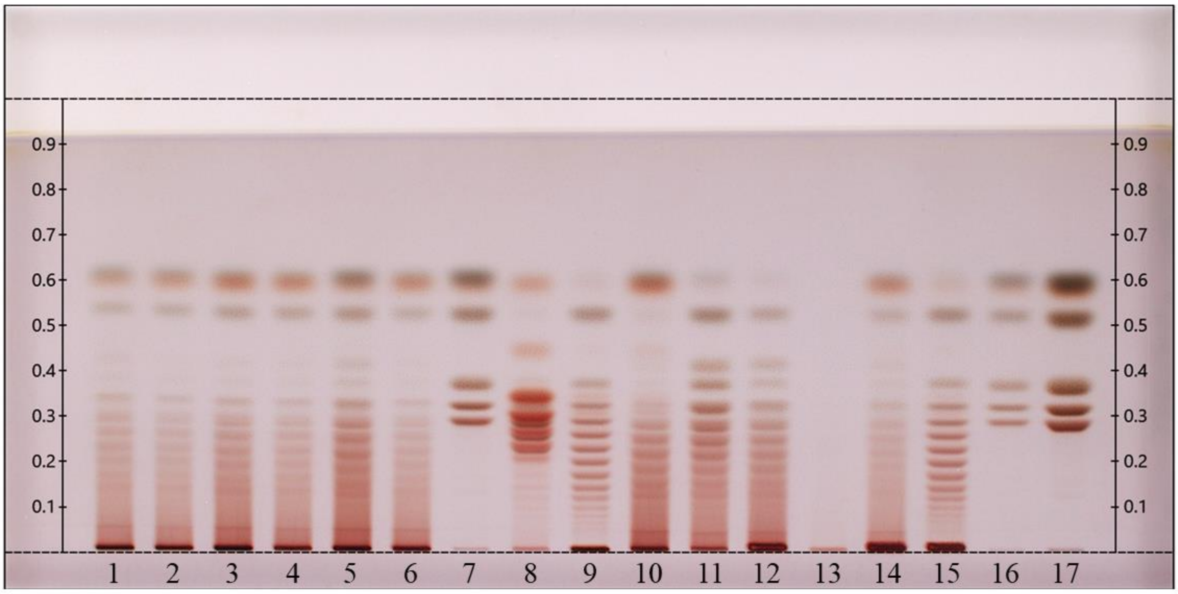

Supplementary Figure S4. High performance thin layer chromatography (HPTLC)-analysis of fructan extracts. Lanes 1 – 6 correspond to *Agave amica* extracts. Lanes 8 – 15 correspond to raftilose, raftiline, *Dasylirion* sp., *Agave angustifolia*, *Agave potatorum*, levan mixture, *Agave tequilana*, and raftiline GR, respectively. Lanes 7, 16, and 15 correspond to a standard compound mixture (from top to bottom) of glucose, fructose, 1-kestose, 1-nystose, and 1-F fructofuranosylnystose at 2 mg/mL with application volumes of 1.0, 0.5, and 1.5 µL, respectively.
